# Profiles of Depressive Symptoms and Anger in Men: Associations With Postpartum Family Functioning

**DOI:** 10.3389/fpsyt.2020.578114

**Published:** 2020-11-23

**Authors:** Jacqui A. Macdonald, Christopher J. Greenwood, Lauren M. Francis, Tessa R. Harrison, Liam G. Graeme, George J. Youssef, Laura Di Manno, Helen Skouteris, Richard Fletcher, Tess Knight, Joanne Williams, Jeannette Milgrom, Craig A. Olsson

**Affiliations:** ^1^Faculty of Health, Centre for Social and Early Emotional Development, School of Psychology, Deakin University, Geelong, VIC, Australia; ^2^Centre for Adolescent Health, Population Health Theme, Murdoch Children's Research Institute, Parkville, VIC, Australia; ^3^Department of Paediatrics, Faculty of Medicine, Dentistry and Health Sciences, University of Melbourne, Parkville, VIC, Australia; ^4^Monash Centre for Health Research and Implementation, School of Public Health and Preventive Medicine, Monash University, Clayton, VIC, Australia; ^5^Warwick Business School, University of Warwick, Coventry, United Kingdom; ^6^Faculty of Health and Medicine, Family Action Centre, University of Newcastle, Callaghan, NSW, Australia; ^7^Cairnmillar Institute, Hawthorn East, VIC, Australia; ^8^Department of Health Sciences and Biostatistics, Swinburne University of Technology, Hawthorn, VIC, Australia; ^9^Parent-Infant Research Institute, Austin Health, Melbourne, VIC, Australia; ^10^Melbourne School of Psychological Sciences, University of Melbourne, Parkville, VIC, Australia

**Keywords:** depression, men, postpartum, co-parenting, bonding, anger, social support, father

## Abstract

**Background:** Evidence suggests that men commonly experience depression as feelings of anger; yet, research has not investigated what this means for the manifestation of depressive symptoms in the early years of fatherhood and for key indicators of family functioning.

**Methods:** Using data from a longitudinal cohort study of men at the normative age for entering fatherhood (28–32 years), we conducted latent class analyses to identify patterns of depressive symptoms and 3 sub-types of state anger (feeling; verbal; physical). We then assessed whether class membership was associated with paternity status (*n* = 535). In a subsample of fathers of infants aged up to 18 months (*n* = 162), we prospectively assessed associations with paternal-infant bonding, co-parenting, perceived social support, paternal involvement in childcare and alcohol use up to 2 years later.

**Results:** Five classes emerged that differentiated men by anger and depressive symptom severity and by the degree to which men endorsed the feeling of wanting to express anger physically. Compared to the reference class with minimal symptoms, fathers had a higher probability of being in either the mild or most severe symptom classes. Men in symptomatic classes were at higher risk of lower levels of social support, co-parenting problems, and paternal-infant bonds. Class membership was not associated with alcohol use or paternal involvement in childcare.

**Conclusions:** Our results reveal patterns of co-existing symptoms of depression and anger in fathers of infants that will be relevant to men's own need for support, their family safety, partner mental health and child developmental outcomes.

## Introduction

Approximately one in ten fathers of infant children experience depression, with prevalence peaking at 25% between 3- and 6-months postpartum (1, 2). Paternal depression is associated with alcohol misuse (3), lower social support (4), poorer quality father-infant bonds (5–7), and higher risk of partner relationship problems (8), overall indicating an environment of increased physical and emotional risk for the father, his partner and the developing child (9). Compared to non-depressed fathers, those reporting depressive symptoms have poorer interactions with their infant children, including higher frequency of spanking (10, 11). Effects of paternal depression on children are also evident over time (12). In prospective longitudinal studies, infants of depressed fathers are at increased risk for developmental difficulties (13) and behavioral and emotional problems up to 7 years of age, even after accounting for maternal depression (14, 15). Most perinatal paternal depression is preceded by a history of pre-conception depression (16, 17) and so research in this area informs both prevention and intervention.

Gender differences in depression are widely reported [See (18–20)]. They are evident in consistently higher rates of depression diagnosis in females (21), who also, on average, experience earlier symptom onset than males (22), and higher prevalence rates continuing into older age (23). Sex differences are also apparent in the genetics and neurobiology of depression including differential dysfunction detected in prefrontal neuronal circuitry (24), distinct sex patterns in genetic transcription (25), and varying sex hormone modulation of neurotransmitter systems (26, 27). Evidence exists for both overlapping and distinct male and female symptom presentations of depression with men more inclined than women to report higher rates of risk taking, substance use and anger (28). Compared to women, men are less likely to endorse traditional symptoms of depression including sadness and crying (29, 30). Debate now exists over whether the different symptom manifestations have resulted in under-diagnosis of men with depression (31, 32).

Proposed male depression sub-types have been variously coined Masculine Depression (31), Male Depression (33, 34), Masked Depression (35) and Male Depressive Syndrome (36, 37). Common to these is the proposition that exposure to strict gender role socialization increases the likelihood of men minimizing traditionally overt symptoms of depression such as sadness, and instead endorsing sanctioned masculine responses to stress and vulnerability such as self-medicating with alcohol and other substances and venting of anger (38). Masculine norms that promote competition, dominance, aggression, evasion of femininity and stoicism are argued to both increase risk for mental health problems and reduce the likelihood of endorsement of symptoms of depression such as sadness and hopelessness (29, 39–41). Rigid conformity to traditional masculinity is also associated with a lesser tendency to seek help for emotional and psychological needs (42) and with increased suicide risk (43). Proponents of a male depression sub-type therefore argue that screening men for emotion such as anger may improve detection of depression and in turn increase opportunities for treatment (41, 44).

Empirical evidence supports the proposition that for many men anger accompanies depression. For example, in patients diagnosed with Major Depressive Disorder, compared to women, men were twice as likely to experience anger attacks during depressive episodes (45). In a community sample of men (*n* = 499), anger was correlated with depressive symptoms (*r* =0.57) with the effect strongest in those who identified with extreme masculine gender norms (33). In a sample of 102 men who had experienced a stressful life event, a moderate correlation was reported between depression and externalizing (*r* = 0.32) indicated by 11 items including having a “short fuse,” punching something, or yelling at someone.

Importantly, it is not that women do not experience anger in the context of depression because research indicates they do (40, 46). Instead, the contention is that observable symptoms align with gender-based socialized expectancies whereas current diagnostic criteria and screening instruments are weighted toward to female symptom presentations (33, 44). Regardless of gender differences, at the population level there is normative variation within gender for levels of felt anger intensity, and in expression from constraint to overt displays of verbal and physical anger (47). Such variation suggests the potential for heterogeneity among men in how anger might align with symptoms of depression.

To date the research that has examined depression and anger in men has focused heavily on gender differences or associations between variables. These variable-centered analytic approaches assume homogeneity in men in the way they experience and express depressive symptoms (48). In contrast, person-centered analytic approaches such as latent class or profile analysis detect heterogeneous profiles within a population, revealing variation in symptom presentations that may be relevant to assessment, diagnosis and treatment.

Only one prior study in a community-based non-clinical sample has sought to understand this heterogeneity in men with regard to depression and anger (49). It explored latent profiles of young Canadian men aged 18–25 years based on internalized depressive symptoms (e.g., feeling down and hopeless) and externalized symptoms, specifically, anger and aggression, drug use, alcohol use and risk-taking. Three distinct classes were found classifying men as high externalizing, high internalizing and asymptomatic. The externalizing group had the highest incidence of a recent suicide plan or attempt. However, in this study high drug use was a defining characteristic of the externalizing group and may well have driven the class differentiation. Drug use may stimulate anger (50) and precede depression (51) and so may confound understanding of associations between the two. How men's feelings of anger coalesce with current definitions of depression (regardless of substance intake) remains unclear. The emotional state of anger does not always have an externalized expression and yet may still be a useful indicator of mental health risk (52). Additionally, developmentally, in the prior latent class analysis (LCA), the Canadian men spanned late adolescence to emerging adulthood and so profiles represented a life stage in which experimentation with drugs and risk-taking behaviors may be at their peak (53, 54). Prominent characteristics of young men are likely tempered in slightly older men as they take on demands and responsibilities of adulthood at the normative age for a transition to fatherhood (55).

In the context of men who are fathers, understanding the role of anger in the presence of paternal depressive symptoms provides information about risk in the home to the partner and infant. It potentially informs paternal mental health screening questions and it may inform discourse around factors that contribute to family safety and emotional security. The transition to fatherhood has been identified as a period of heightened risk for mental health problems and stress (1, 56–58) and paternity status may itself play a role in the manner in which depression and anger co-occur. Changes to paternal levels of testosterone across the transition to fatherhood may also be a factor in levels of anger and depressive symptoms (59, 60). A large cohort study of men (*n* = 624) found that those with naturally higher levels of testosterone were more likely to become fathers, and experience a drop in testosterone in the first year postpartum (59). Low testosterone levels have been linked to increased risk of depressive symptoms (61) specifically for first time fathers (60). Higher testosterone has been linked with increased externalizing symptoms such as antisocial behavior (61) and intimate partner violence in new families (60).

The purpose of this study was to examine relationships between depressive symptoms and anger in fathers of infants using rare Australian data from the Men and Parenting Pathways (MAPP) cohort study. Specifically, the aims were to: (1) identify latent classes of men at the normative age for first-time parenthood (28–32 years) that differentiate patterns of depressive symptoms and anger; (2) assess whether class membership is associated with paternity status; and, (3) in a subsample of fathers of infants, investigate associations between fathers' class membership and indicators of family functioning including the quality of the father-infant bond, the co-parenting relationship, paternal involvement in childcare, paternal social support and paternal alcohol misuse.

## Methods

### Participants and Procedure

Men and Parenting Pathways (MAPP) is a national cohort study of Australian resident males at the peak age for first-time fatherhood (62). Over a 2-year period beginning in February 2015, men aged 28–32 years (both fathers and non-fathers) were recruited via social and traditional media, organizational partners and word of mouth. At baseline, 608 men (Mean age = 29.86 years, SD = 1.33) provided online consent to complete five annual web-surveys assessing a range of domains including demographics, mental health and well-being, workplace stress, family and peer relationships. Ethics approval was granted by the Deakin University Faculty of Health, Human Research Ethics Committee project number HEAG-H-192-2014.

At the time of analysis, three waves of data had been completed with surveys 4 and 5 simultaneously ongoing. Surveys are completed in REDCap secure web-based software platform (Research Electronic Data Capture) (63). One year following each survey completion, participants are sent an automatic email invitation to the next wave survey. If they do not complete the survey after one reminder email, a number of engagement strategies are implemented. These include a selection of phone-calls, SMS messages, Facebook messages, or mailed letters, depending on participants' nominated preferences. Participants who complete their annual survey are entered in a prize draw to win a $AUD100 voucher for a retail store (usually a hardware or grocery chain). These strategies have resulted in a participation rate of 83% of the original sample completing surveys at either wave 2 or 3. The MAPP cohort reflects the Australian population with regard to participants living in areas of relative advantage and disadvantage (64). Participants are slightly more likely than men of the same age in the population to have completed year 12 education (65) and to be in paid employment (66). The cohort is mostly Australian born (88%) compared to Australian men of the same age (62%) (67) but is representative of Indigenous Australian and Torres Strait Islanders (68) and slightly over-representative of non-heterosexual men (69). See Macdonald et al. (Open Science Framework) for additional cohort profile information (70).

The current study was completed in two stages. To be included in the first stage, men had to provide data at wave 1 on both depressive symptoms and state anger (*n* = 535). The majority of these men were Australian-born (87.8%), had achieved an education at higher than year 12 (78.3%), and identified as heterosexual (92.4%).

The subsample of men included at the second stage were those included in stage 1 who were fathers either of an infant aged 12 months or younger at wave 1 or an infant aged 18 months or younger at waves 2 or 3 (*n* = 162). The older infant age range in waves 2 and 3 ensured father-infant data were collected even if men were delayed in completing their annual survey.

### Measures

#### Time 1 Latent Class Indicators

##### Symptoms of depression

The Depression Anxiety Stress Scale 21 (DASS-21) (71), seven-item subscale was used to assess depressive symptoms at Wave 1. Participants were asked to respond to statements such as “I felt sad and depressed” and “I couldn't seem to experience any positive feeling at all,” on a 4-point Likert scale ranging from 0 = Never to 3 = Almost always, indicating how relevant these statements were to them over the past week. A total score was calculated and then doubled, as per validated scoring instructions, to align with the DASS-42 for which normed data are available (72). Higher scores indicate more severe symptoms. Construct validity of the DASS-21 has been demonstrated in multiple non-clinical samples [e.g., (73, 74)] and as a routine clinical screening instrument (75). In the current sample, internal consistency for this scale was high (α = 0.93).

##### State anger

State anger was measured at baseline using the three subscales of the 15-item state anger scale of the State and Trait Anger Expression Inventory-2 [STAXI-2; (47)]. Participants were presented with statements such as “I felt furious” and “I felt like pounding somebody” and asked to select the response that best described how they felt recently on a four-point Likert scale ranging from “1 = Not at all” to “4 = Very much so.” The 15 items were equally divided across the subscales measuring feeling angry (Feeling), feeling like expressing anger verbally (Verbal) and feeling like expressing anger physically (Physical). A total score was created for each set of five items pertaining to their subscale, with higher scores indicating higher levels of anger. Internal consistency for the items within these subscales was high (α_Feeling_ = 0.93, α_Verbal_ = 0.91, α_Physical_ = 0.91).

#### Outcomes Indicating the Father-Infant Context

For each participant, outcome data were analyzed only from a single wave in which they had a child within the eligible age range. If fathers had children in the eligible age range at more than one wave, we prioritized inclusion of outcomes measured at wave 2 then wave 3. We did this because our aim was to longitudinally assess associations between class membership at wave 1 and the subsequent parenting-infant context outcomes. However, to maximize sample size, we also included men who only had an infant in the eligible age range at wave 1 and used cross-sectional data from that timepoint.

##### Father-infant bonding

Father-infant bonding was measured at each wave with the 19-item Paternal Postnatal Attachment Scale (PPAS; 5). In Wave 1 it was reported on by fathers with an infant up to and including 12 months of age. At subsequent waves, it was reported on by fathers of infants up to and including 18 months of age. The PPAS assesses paternal patience and tolerance, pleasure in interactions with the infant and affection and pride felt toward the infant. Example items include: “When I am caring for the baby, I get feelings that the child is deliberately being difficult or trying to upset me (reversed)” and “When I am with the baby and other people are present, I feel proud of the baby.” Response options are item specific with 2, 3, 4, and 5-point options. Total scores range from 19 to 95 with higher scores indicating higher levels of father-child bonding. The PPAS has been validated in samples of fathers of infants up to 24 months and shown to be negatively associated with paternal depression and parenting stress and positively associated with positive affect (5, 6, 76). In the current study internal consistency was α = 0.82.

##### Co-parenting

At Waves 1 to 3, fathers completed the 12-item Co-parenting Relationship Scale (CRS; 77]. Example item: “My partner and I have the same goals for our child.” Responses are measured on a 7-point Likert scale from 0 = Not true of us to 6 = Very true of us. High scores indicate high levels of agreement, closeness and mutual support with respect to parenting. Validation of the CRS was carried out in a sample of 152 couples when their children were 6 months, 1 year and 3 years with validated relationship scales measuring love, conflict, sex and romance among other constructs (77). Internal consistency in the current study was α = 0.85.

##### Paternal involvement in childcare

Fathers' involvement in childcare was measured at waves 1 to 3 with a six-item scale (78) in which fathers were asked to indicate how often they completed the following activities with their child: feeding, nappy changing, bathing, putting the child to sleep, playing and taking them for a walk. High scores indicate higher frequency of engagement in normative parenting tasks. Responses were measured on a 4-point Likert scale: 0 = Not at all, 1 = Rarely, 2 = Sometimes, 3 = Very often. Possible total scores range from 0 to 18. Internal consistency in the current study α = 0.83.

##### Social support

At each wave, participants self-reported their perceptions of receiving support on the 12-item Multidimensional Scale of Perceived Social Support [MSPSS; (79)]. The scale assesses perceived support from a significant other, friends and family. Example items include, “I get the emotional help and support I need from my family” and “There is a special person who is around when I am in need.” Reponses are measured on a 7-point Likert scale from 1 = Very strongly disagree to 7 = Very strongly agree. Total scores were calculated with higher scores indicating greater perceived support. The scale is widely used and found to be negatively associated with depressive symptoms in men (80). Internal consistency in the current study α = 0.91.

##### Alcohol use

Alcohol use was measured at each wave with the AUDIT-C, a brief version of the Alcohol Use Disorders Identification Test (81). This brief measure has been shown to accurately identify hazardous/risky alcohol use behaviors (82, 83). All participants were asked the item “How often do you have a drink containing alcohol?” with responses; 0 = “Never,” 1 = “Monthly or less,” 2 = “2–4 times a month,” 3 = “2–3 times a week,” 4 = “4 or more times a week.” Participants who indicated a score of 2 or higher were asked the following items: “How many standard drinks containing alcohol do you have on a typical day that you drink alcohol” (Response options: 0 = “1 or 2,” 1 = “3 or 4,” 2 = “5 or 6,” 3 = “7 to 9,” and 4 = “10 or more”) and “How often do you have six or more drinks on one occasion?” (Response options: 0 = “Never,” 1 = “Less than monthly,” 2 = “Monthly,” 3 = “Weekly,” 4 = “Daily or almost daily”). Total scores range from 0 to 12.

##### Potential confounders

Adjustments were made for participant country of birth (0 = Australia, 1 = other), education level (0 = “> year 12,” 1 = “year 12 or less”), household weekly income (0 = “≥ $AUD1,150 weekly,” 1 = “ < $1,150 weekly”), and infant age and sex. Analyses additionally included the adjustment for the wave (1, 2, or 3) at which the outcome indicator of the father-infant context was measured.

### Data Analysis

To identify classes of individuals across depressive symptoms and state anger, a series of latent class analyses (LCAs) were conducted in Mplus v8 (84). In the full sample, LCAs were run with increasing number of classes (from 2 to 6). Optimal class selection was based on key indicators of goodness-of-fit, including Vuong-Lo-Mendell-Rubin (VLMR) and Lo-Mendell-Rubin (LMR) likelihood ratio test *p*-values, lower Akaike information criterion (AIC) and Bayesian information criterion (BIC) values, as well as on entropy values (0.80 or above), and parsimony. The most-likely class membership was extracted from the selected class-solution for subsequent analyses.

All other analyses were conducted in Stata v16 (85). First, to describe the LCA solution, we conducted unadjusted linear regression models in which the depression score and three anger variables were each regressed onto the class membership. Pairwise comparisons across all levels of class membership were conducted to test for differences in depression and anger scores between classes. Second, we conducted an unadjusted logistic regression to examine differences in parent status (father or not) between classes.

Finally, in the subsample of fathers of infants, we conducted a series of linear regression analyses to examine relationships between latent classes and indicators of the father-infant context. Specifically, a series of unadjusted and adjusted models were fit in which each primary outcome variable (i.e., paternal-infant bonding, paternal involvement in childcare, co-parenting, alcohol use and perceived social support) was regressed onto latent class membership.

Multiple imputation was used to handle missing data for the parenting context analyses. Specifically, 20 imputed complete datasets were created, based on a multivariate normal model (86). Binary variables were imputed as continuous variables and then back transformed with adaptive rounding following imputation. Estimates were obtained by averaging results across the 20 imputed datasets with inferences under multiple imputation obtained using Rubin's rules (87).

## Results

### Descriptives

**Figure 1:**
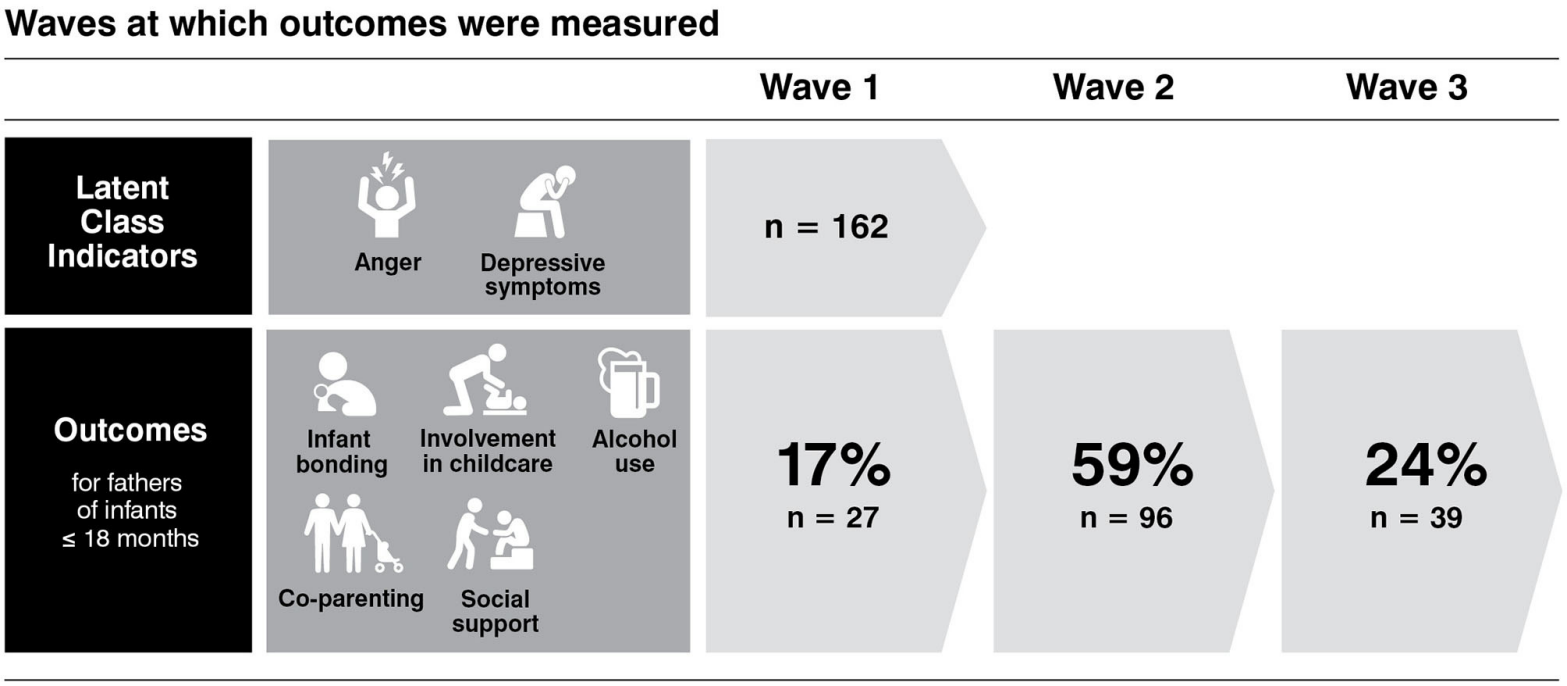
For 83% of subsample participants, the outcome data were assessed in waves subsequent to the assessment of the LCA indicators, anger and depressive symptoms. For the remainder of the subsample (17%), class indicators and outcomes were assessed cross-sectionally.

Table 1 presents proportions of key characteristics for participants in both the total sample, and the subsample of fathers of infants. At wave 1, 39% of men were fathers. For the subsample of fathers with an infant in the eligible age range, Figure 1 shows that for 83% of participants, the outcome data were assessed in waves subsequent to the assessment of the LCA indicators, anger and depressive symptoms. For the remainder of the subsample (17%), class indicators and outcomes were assessed cross-sectionally. Infant children in the subsample were 49% female with a mean age of 7.5 months (SD = 4.5 months). In the subsample, 46.3% of participants were first-time fathers. For one father, the focal child was a step-child, all others were biological children. Mean scores for outcome variables were: postpartum bonding, 74.48 (SD = 9.13); paternal involvement in childcare, 14.08 (SD = 3.44); co-parenting, 57.61 (SD = 10.94); AUDIT-C, 4.75 (SD = 2.50); and social support, 64.82 (12.89).

**Table 1:** Demographic characteristics of men included in the latent class analysis (LCA; *n* = 535) and the sub-sample of fathers of infants (*n* = 162).

	**LCA Sample**		**Father of infants**	
	**%**	**95% CI**	**%**	**95% CI**
Birth outside Australia	12.20	(9.67, 15.26)	12.96	(8.60, 19.08)
Education > Year 12	21.68	(18.39, 25.39)	14.81	(10.12, 21.17)
Identify as not heterosexual	7.56	(5.57, 10.19)	1.25	(0.31, 4.87)
Income < $1,150 AUD weekly	25.42	(21.9, 29.29)	11.11	(7.11, 16.96)

### Latent Class Analysis (LCA): Depression and Anger

**Figure 2:**
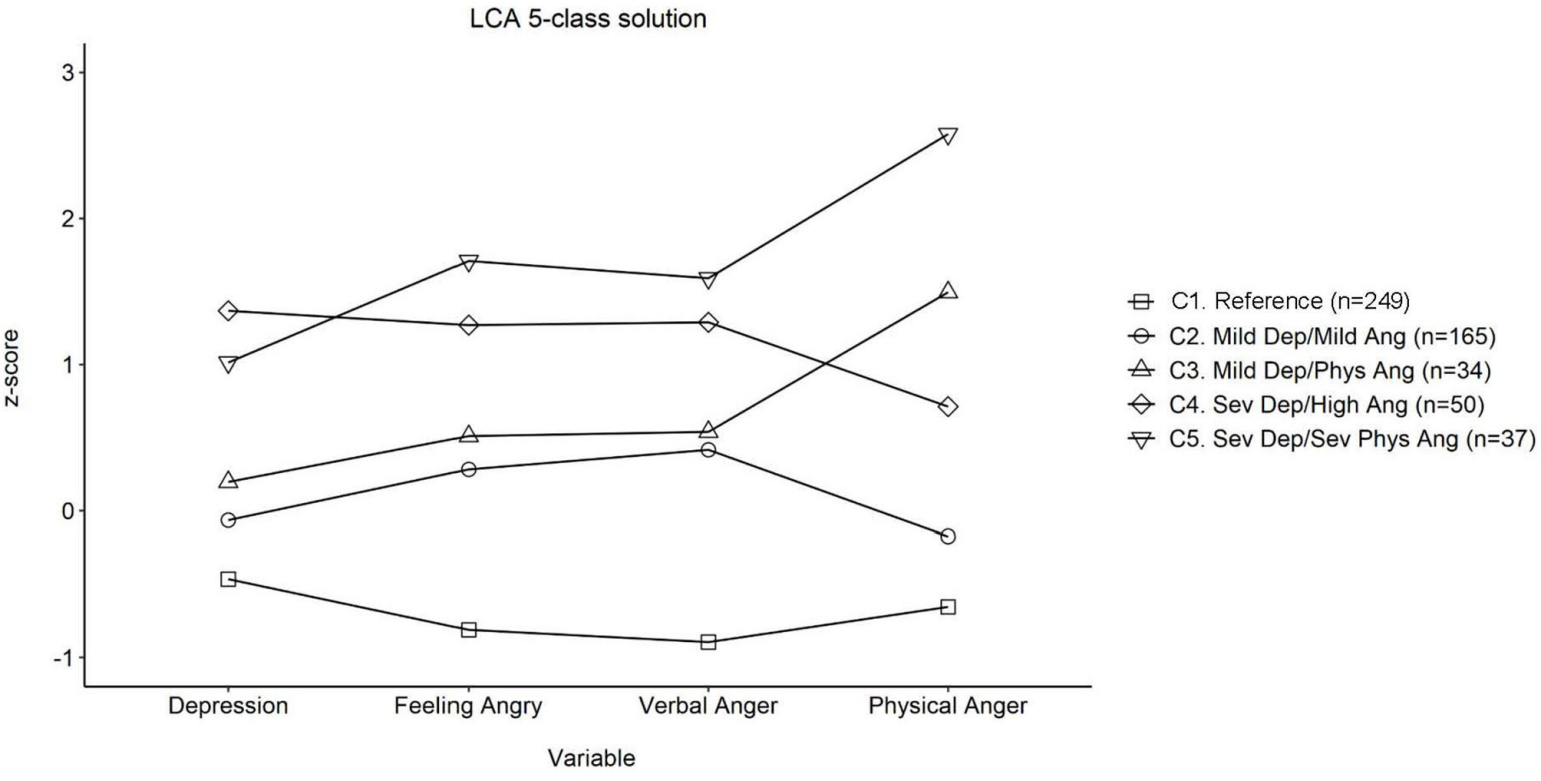
Standardized means for symptoms of depression and anger within five latent classes found in men aged 28–32 years. Dep, Depression; Ang, Anger; Phys, Physical; Sev, Severe. The mean of our sample (*z* = 0) sat within the DASS-21 designated mild category of depressive symptoms. At one SD above the mean, the sample sat in the DASS-21 severe category (71).

Fit statistics for the LCA are presented in Table 2. The LMR and VLMR *p*-values suggested that a 2-class solution fit better than a 1-class solution. Although *p*-values did not suggest that the 3-class solution fit better than the 2-class solution, BIC and AIC values still showed a decline from the 2-class to 3-class solution. Following this, subsequent 3 to 5-class solutions showed improvement on the previous solution. The 6-class solution, however, did not show better fit (based on LMR and VLMR *p*-values) than the 5-class solution. The selected 5-class solution, presenting differences between groups in standardized values for each indicator, is presented in Figure 2. By comparison, Table 3 presents means and 95% CIs of the unstandardized depression and state anger subscales for the selected five-class LCA model, alongside pairwise mean comparisons for each construct.

**Table 2:** Model fit indices for latent classes: 2- to 6-class solutions.

	**BIC**	**AIC**	**LMR**	**VLMR**	**Entropy**
2-class	5250.941	5195.271	<0.001	<0.001	0.869
3-class	4919.449	4842.368	0.125	0.119	0.866
4-class	4775.567	4677.075	0.004	0.003	0.879
5-class	4701.619	4581.715	0.015	0.013	0.892
6-class	4623.142	4481.827	0.086	0.079	0.889

**Table 3:** Means and 95% confidence intervals of depression and state anger sub-scales for each of the five latent classes, and significant differences between classes (*n* = 535).

**Construct**	**C1. Reference**		**C2. Mild Dep/Mild Ang**		**C3. Mild Dep/Phys Ang**		**C4. Sev Dep/High Ang**		**C5. Sev Dep/Sev Phys Ang**		**Sig. contrasts**
	***M***	**95% CI**	***M***	**95% CI**	***M***	**95% CI**	***M***	**95% CI**	***M***	**95% CI**	***p* <0.05**
Depression	6.48	(5.46, 7.51)	10.62	(9.36, 11.87)	13.00	(10.23, 15.77)	26.25	(23.97, 28.53)	21.63	(18.98, 24.28)	1 <2, 3, 4, 5; 2 <4, 5; 3 <4, 5; 5 <4
S/Ang-F	8.97	(8.69, 9.24)	13.59	(13.26, 13.93)	14.18	(13.43, 14.92)	17.68	(17.07, 18.29)	19.32	(18.61, 20.04)	1 <2, 3, 4, 5; 2 <4, 5; 3 <4, 5; 4 <5
S/Ang-V	8.46	(8.20, 8.72)	14.66	(14.34, 14.98)	14.84	(14.13, 15.54)	18.42	(17.84, 19.00)	19.73	(19.06, 20.40)	1 <2, 3, 4, 5; 2 <4, 5; 3 <4, 5; 4 <5
S/Ang-P	5.53	(5.35, 5.72)	7.50	(7.28, 7.73)	14.35	(13.86, 14.85)	11.17	(10.77, 11.58)	18.57	(18.09, 19.04)	1 <2, 3, 4, 5; 2 <3, 4, 5; 3 > 4; 3 <5; 4 <5

*Dep, Depression; Ang, Anger; Phys, Physical; Sev, Severe; Sig, significant. C1, Reference class; C2 class has mild depressive symptoms and mild levels of anger on all 3 subscales; C3 class has mild depressive symptoms, mild generalized anger and verbal anger and severe levels of physical symptoms; C4 class has severe levels of depressive symptoms and high levels of anger on all 3 subscales; C5 class has severe levels of depressive symptoms and high levels of generalized and verbal anger and severe levels of physical anger*.

Comparisons demonstrate that across all measures of depressive symptoms and state anger, participants in the “Reference” class (Class 1; C1) scored lower than all other classes. The mean score for depressive symptoms for C1 was in the “normal” range for DASS classifications of symptom severity (71). Participants in Class 2 (C2), the “Mild Depression/Mild Anger” symptoms class, demonstrated lower depression, and lower anger feelings and verbal anger than those in Class 4 (C4) “Severe Depression/High Anger” and Class 5 (C5) “Severe Depression/Severe Physical Anger.” On expressing anger physically, C2 “Mild Depression/Mild Anger” was higher than C4 “Severe Depression/High Anger” but lower than the C5 “Severe Depression/Severe Physical Anger.” Participants in Class 3 (C3), “Mild Depression/Physical Anger,” scored lower across all measures of depression and state anger then those in C4 “Severe Depression/High Anger” and C5 “Severe Depression/Severe Physical Anger.” Similarly, participants in the C4 “Severe Depression/High Anger” scored lower across all measures of depression and state anger then those in C5 “Severe Depression/Severe Physical Anger”. Mean depressive symptoms scores for C2 and C3 were within the DASS-21 mild symptoms range whereas scores for C4 and C5 were in the severe depressive symptoms range (71).

### Class Membership and Parent Status

Compared to men in the C1 Reference group (31% fathers), there was a greater proportion of fathers (vs. non-fathers at Wave 1) in the C2 Mild Symptom class (48% fathers; OR = 2.05, 95% CI [1.37, 3.08]) and in the C5 Most Severe Symptom class (49% fathers; OR = 2.12, 95% CI [1.05, 4.25]). There was little evidence to suggest the proportion of fathers was different between the C1 and C3 (44% fathers) or C4 (40% fathers) classes (OR = 1.49, 95% CI [0.80, 2.79] and OR = 1.76, 95% CI [0.85, 3.65], respectively).

### Class Membership and Parenting Context Outcomes

Within the subsample of fathers of infants, 49% were in the C1 Reference class (*n* = 80), 33% were in the C2 “Mild Depression /Mild Anger” class (*n* = 54), 6% were in the C3 “Mild Depression/Physical Anger” class (n = 9), 6% were in the C4 “Severe Depression/High Anger” class (*n* = 10), and 6% were in the C5 “Severe Depression/Severe Physical Anger” class (*n* = 9).

Table 4 presents the unadjusted and adjusted results from the linear regression analyses for which each of the outcome variables (Paternal Postpartum bonding, Paternal Involvement in childcare, Co-parenting, Alcohol use, Perceived Social Support) were regressed onto the depression/anger classes.

**Table 4:** Unadjusted and adjusted unstandardized effects and 95% confidence intervals (CIs) for regression analyses assessing father-infant context variables predicted by latent class membership.

	**Unadjusted**		**Adjusted**	
**C1. Reference group**	***b***	**95% CI**	***b***	**95% CI**
	**Paternal postpartum bonding**			
C2. Mild Dep/Mild Ang	−1.04	(−4.06, 1.98)	−1.30	(−4.48, 1.87)
C3. Mild Dep/Phys Ang	−8.96	(−18.59, 0.68)	−9.14	(−18.58, 0.31)
C4. Sev Dep/High Ang	−3.88	(−7.86, 0.10)	−3.93	(−8.48, 0.62)
C5. Sev Dep/Sev Phys Ang	**−6.10**	**(−11.84**, **−0.36)**	**−7.76**	**(−14.22**, **−1.29)**
	**Paternal involvement in childcare**			
C2. Mild Dep/Mild Ang	0.44	(−0.81, 1.68)	0.87	(−0.37, 2.11)
C3. Mild Dep/Phys Ang	−1.67	(−4.22, 0.88)	−1.92	(−4.31, 0.46)
C4. Sev Dep/High Ang	−0.68	(−3.14, 1.77)	−0.38	(−2.74, 1.98)
C5. Sev Dep/Sev Phys Ang	1.09	(−0.45, 2.64)	0.97	(−0.72, 2.67)
	**Co-parenting**			
C2. Mild Dep/Mild Ang	**−4.40**	**(−8.19**, **−0.61)**	**−5.46**	**(−9.51**, **−1.41)**
C3. Mild Dep/Phys Ang	−7.23	(−19.71, 5.26)	−7.80	(−19.92, 4.31)
C4. Sev Dep/High Ang	**−12.33**	**(−18.93**, **−5.73)**	**−14.05**	**(−21.05**, **−7.06)**
C5. Sev Dep/Sev Phys Ang	**−6.06**	**(−11.53**, **−0.59)**	**−8.56**	**(−15.00**, **−2.12)**
	**Alcohol use**			
C2. Mild Dep/Mild Ang	0.52	(−0.39, 1.42)	0.26	(−0.65, 1.18)
C3. Mild Dep/Phys Ang	2.08	(−0.02, 4.18)	1.77	(−0.24, 3.77)
C4. Sev Dep/High Ang	−0.22	(−1.86, 1.42)	−0.16	(−2.05, 1.72)
C5. Sev Dep/Sev Phys Ang	−0.09	(−1.77, 1.59)	−0.10	(−1.79, 1.59)
	**Perceived social support**			
C2. Mild Dep/Mild Ang	**−4.51**	**(−8.39**, **−0.62)**	**−4.14**	**(−8.29, 0.00)**
C3. Mild Dep/Phys Ang	**−12.49**	**(−22.48**, **−2.50)**	**−12.16**	**(−21.81**, **−2.50)**
C4. Sev Dep/High Ang	**−8.33**	**(−14.96**, **−1.71)**	**−9.63**	**(−17.50**, **−1.76)**
C5. Sev Dep/Sev Phys Ang	**−16.00**	**(−29.86**, **−2.14)**	**−17.42**	**(−32.96**, **−1.88)**

*Adjusted analyses are adjusted for wave, birthplace, education, household income, child age and child sex; effects significant (in bold) when CIs do not overlap zero. C1–C5 (Class 1 to Class 5); Dep, Depression; Ang, Anger; Phys, Physical; Sev, Severe*.

Compared to the C1 Reference class, only fathers in C5 “Severe Depression/Severe Physical Anger” clearly reported lower Paternal Postpartum bonding although weak evidence was found also for poorer bonds felt by fathers in C3 “Mild Depression/Physical Anger.” Findings did not suggest differences between the Reference class and any other class on Paternal Involvement in childcare or Alcohol use. Co-parenting scores were lower for participants in the C2 “Mild Depression/Mild Anger,” C4 “Severe Depression/High Anger,” and C5 “Severe Depression/Severe Physical Anger” classes compared to the C1 Reference class. Finally, Perceived Social Support scores were lower for fathers in all classes compared to the C1 Reference class.

To determine if the results could be interpreted as longitudinal findings, we ran analyses again removing fathers with only wave 1 outcome data. For all outcomes, directions of the associations were replicated but with less precision in estimates, presumably because of the smaller sample. We therefore elected to report results for the full sample of fathers as described from waves 1 to 3.

## Discussion

We identified profiles of depressive symptoms and anger in men at the normative age for becoming a father. We found five groups (C1–C5) that differentiated men by their symptom severity and by the degree to which they experienced the emotion of anger as a desire to express anger physically. We then found that compared to the C1 Reference group, there was a higher probability of being a father in the C2 mild symptoms group and the C5 severe symptoms group with the most elevated physical expression of anger. We then investigated effects of symptom profiles on a subsample of men who had an infant or who went on to have an infant child in the next 2 years. Compared to the Reference group fathers, those in all four symptomatic groups reported significantly lower levels of perceived social support. Furthermore, compared to the Reference group, fathers in three of the symptomatic groups reported significantly poorer co-parenting relationships with their partners. Only fathers with the most severe symptoms of both depression and physical anger reported significantly poorer bonding with their infant child. Group membership did not predict paternal involvement in childcare or alcohol use.

The classes that emerged in our sample indicate that feelings of anger coexist with depressive symptoms in men and that the degree of felt anger corresponds to the severity of depression. This finding aligns with arguments that support inclusion of anger as a diagnostic indicator of male depression (33, 34). Our study extends understanding of this relationship by showing a divergence in how anger is felt at different levels of depressive symptoms. This is best represented visually by the forks in Figure 2 that split those with mild and severe depressive symptoms into groups with either higher or lower levels of feeling their anger as a desire to lash out physically. The items in the physical anger subscale refer to breaking or banging objects and to “kicking,” “hitting” and “pounding” a person and are indicative of risk to family safety.

In prior research stronger feelings of anger were reported in men who engaged in marital violence compared to those who did not (88). In our sample, almost one in four men reported elevated levels of physical feelings of anger. Even with mild depressive symptoms, men in the C3 “Mild Depression/Physical Anger” group, reported more intense feelings of physical anger than the C4 “Severe Depression/High Anger” group. In the family context, these diverging patterns of desire for physical expression of anger are likely to be important markers of paternal emotional state as well as risk to maternal mental health, child development outcomes, and family safety.

Our focus was on the relevance of depressive symptom and anger profiles for men with infant children or men likely to soon become fathers. We found that a higher proportion of fathers were in the C2 Mild symptom group than in the Reference group. This finding aligns with evidence that fatherhood in the early years is a period of increased emotional vulnerability (1, 2, 56) and suggests the need to ensure that services that interact with families find ways to identify and screen for paternal mental health risk (89). Importantly, for the purposes of perinatal paternal screening, it should be noted that C2 class members endorsed feelings of anger, and of wanting to express anger verbally, at a full standard deviation higher than the reference group. Despite this, C2 men with mild symptoms may be less likely to be identified as at risk because they are less likely to feel like expressing anger physically. We also found that a greater proportion of fathers were in the C5 extreme anger group compared to the Reference group. Possible reasons for this are speculative and warrant further investigation but may be related to findings that that those with naturally higher levels of testosterone are more likely to become fathers, and while most will experience a postpartum drop in testosterone (59), higher testosterone may be associated with externalizing behavior (61) including intimate partner violence in new families (60).

In the subsample of men in our study who were fathers of infants, or who became fathers in the following 2 years after depression and anger were assessed, we found that their symptom profiles were associated with factors relevant to their paternal functioning, particularly in the interpersonal domain. The first clear finding was that compared to the Reference class, all fathers with symptoms of depression and anger from mild to severe were at greater risk of reporting low perceived social support. Our measure of social support asked about support from significant others, friends and family. In line with past research, our finding suggests that many fathers may feel alienated from effective support. In prior research fathers report not seeking support because of reasons including a belief that the focus should be on the mother and infant in the postnatal period (89), past negative experiences when asking for help (90, 91), stigma attached to revealing emotions and vulnerability (92, 93) and rigid adherence to masculine values of stoicism and self-reliance (90, 94).

Fathers with depressive symptoms and anger may also lack motivation to seek support, or may find others are unwilling to provide support because of negative emotional responses when it is offered (95). Depressive symptoms may inform the perception of support and increase the likelihood of negative appraisals of interactions with others (96). Importantly, in our sample, effect sizes for these associations increased as group mean scores on physical anger increased, indicating that the presentation of angry emotion is a clear obstacle to men accessing social support which could be of benefit in managing their depressive symptoms.

We also found that compared to the Reference group, fathers with mild to severe symptoms of depression and anger reported poorer co-parenting relationships with partners. This association was clearly evident for the C2, C4, and C5 groups. A congruent effect size was also apparent for the C3 group; however, confidence intervals around that estimate were wide and so are interpreted as no association. Co-parenting, which describes the cooperation and mutual support between parents in relation to their parenting tasks and decisions, is associated with a range of child developmental outcomes. Specifically, when co-parenting relationships are characterized by disagreement, criticism and conflict, children are at a greater risk of emotional difficulties (97, 98), poorer social competence (99) and lower capacity for theory of mind (100). Our results are in line with previous research demonstrating associations between depressive symptoms and co-parenting (101). A poor co-parenting relationship is also a perpetuating factor in ongoing depression. In a sample of 129 couples assessed from pregnancy to 30 months postpartum, mothers and fathers with high negative interactions experienced steeper increases in depression between 3 and 30 months postpartum (102). Additionally, fathers who reported lower positive interactions experienced an increase in anxiety over the same postpartum period (102). Interventions for families in conflict may benefit from evidence that anger and depressive symptoms potentially co-exist in a bi-directional relationship with co-parenting.

When compared to Reference group fathers, only one group was at clear risk of poorer father-infant emotional bonds. The fathers in the C5 group were those with severe symptoms of depression and the highest scores on physical anger. Brockington (103), whose work involves assessment, diagnosis and treatment of maternal-infant bonding, notes that pathological anger accompanies mother-infant relationship disorders at the extreme end of the spectrum. Brockington's own assessment of parent-infant bonding was designed to screen for risk of infant abuse (104). The scale of father-infant bonding used in the current study captures the related construct of tolerance of the child with questions about annoyance and irritability, impatience and resentment, which may indicate feelings of anger (5). In prior research, fathers who perpetrated intimate partner violence were shown to be less successful in establishing father-infant bonds than those who were not violent (105). However, the current study is the first to demonstrate a link between the paternal-infant bond and co-existing severe depressive symptoms and feelings of anger experienced as the desire to act out physically.

We found no associations between fathers' profiles of depressive symptoms and anger and their alcohol use or involvement in childcare tasks. The finding for alcohol use is somewhat surprising given prior studies of associations between depression and alcohol use (106), and anger and alcohol use in men generally (107). The lack of an association is important to note given previous research that includes alcohol use in latent class analyses to capture male sub-types of depression (49). Our findings are in line with a community-based study of Finnish parents that reported no relationship between alcohol consumption and depressive symptoms in fathers of infants aged 4- to 18-months (108). Similarly, a New Zealand study reported that fathers whose partners were diagnosed with postpartum depression had more symptoms of depression and aggression than men whose partners did not have depression: in line with our results, there were no differences between groups in alcohol use (109). However, conflicting findings exist in the literature with associations between depressive symptoms and alcohol use reported in “at risk” fathers in the U.S. study of Fragile Families and Child Well-being (110) and in a community sample in Brazil (3). In our study, the AUDIT-C mean score indicated risky levels of alcohol use but was below the most accurate cutoff (≥5) for identifying drinking above recommended weekly limits (111) or for detecting diagnosis of alcohol use disorder (112). In Australia, between 38% and 46% of adult men aged 25–39 years, report consuming alcohol above single occasion national risk guidelines (113). In such populations, where normative alcohol consumption by adult males is highly prevalent at levels characterized as “at risk” (114), drinking levels may not be differentiated by the risk indicators in our profiles. Or it may be that fathers who have otherwise healthy mental health profiles may turn to alcohol as a relaxant to adjust to the normative stress of the transition to parenthood (58, 110). In a large analysis of three Australian and New Zealand cohort studies there was little evidence of new fathers reducing pre-conception risky drinking behaviors over the transition to parenthood (115). Prior prospective evidence also suggests that there is a stronger causal relationship between alcohol use and subsequent depression than vice versa (116). In our study we measured depressive symptoms prior to alcohol use, which may also explain the lack of association.

The finding that class membership was not associated with father involvement in childcare was also surprising. In previous research, paternal depression has been found to be negatively associated with father involvement, including time spent together (117); however, measures of this construct vary across studies. In the current study, our questions were entirely task oriented and fathers reported overall higher involvement in tasks such as feeding and clothing infants [*M* = 14.08 (SD = 3.44)] than in the Japanese cohort study in which the measure was originally reported [*M* = 10.04 (SD = 3.2)] (78). It is possible that proximal demands within the home or parental divisions of childcare tasks may be stronger determinants of engagement in these types of tasks than symptoms of emotional health (118). It is also possible that responses were biased by social desirability given growing expectations for a balanced division of parenting duties (119).

The current study has a number of strengths and limitations. A strength was the availability of longitudinal data on preconception and perinatal fathers given that paternal mental health has gained considerably less attention in research than maternal mental health. While the subsample of fathers used to examine family adjustment outcomes included a small number of men who reported outcomes concurrent with LCA measures, we found no interpretative differences in results when these men were excluded from the analyses. Therefore, the current results can be interpreted as prospective findings demonstrating longitudinal associations between men's patterns of depression and anger on paternal outcomes, 1 to 2 years later. This suggests a potential window for either pre-conception or antenatal intervention with fathers experiencing these symptoms.

A high proportion of our sample reported depressive symptom severity above the “normal” range, suggesting our sample are at higher risk than the general population. A Swedish study of 447 fathers similarly found high rates of depressive symptoms (120). Both our study and the Swedish study recruited participants largely online where men at higher risk of mental health issues are potentially more likely to self-select (121, 122). Notwithstanding, we did not aim to recruit a representative sample. Our study focus is not on prevalence, but rather on understanding risk relationships and so, for our purposes, sampling men at risk of psychosocial problems increases our capacity to detect associations of interest.

As is common in longitudinal studies, we experienced some level of attrition; however, our retention of 83% of participants across at least two waves is comparatively high when compared to other cohort studies of men (123). We also used multiple imputation to minimize biases introduced by participants who missed a wave or were lost to follow-up. Another retention strategy was to limit the length of our annual surveys. This necessitated the use of short-form self-report screening instruments. We selected measures widely used and validated in epidemiological studies but acknowledge that these can lack the precision of clinical diagnostic instruments and interviews.

The current research has implications for clinicians working with men generally and with future and current fathers of infants. We present clear evidence of patterns of depressive symptoms and anger in men providing insight into possible manifestations of male emotional vulnerability. This evidence is important because not only do some men minimize or not recognize their own symptoms of depression but clinicians may also misinterpret indicators of risk because of their own gender biases (42, 44). In the context of perinatal care, some health care practitioners have reported apprehension about engaging with fathers because of the possibility of being met with anger (124). Practitioners who have trained specifically in paternal engagement have higher levels of confidence in their capacity to interact with fathers (125). If anger is a common symptom of men's depression and depression is a risk to partner mental health and child development, then widespread practitioner training is warranted for engaging fathers who feel anger.

It is also important to acknowledge that fathers in this sample whose anger was experienced as a desire to be physically aggressive were the minority. A desire to express anger verbally was more common and if acted upon in the family home may be experienced by partners and children as threatening and emotionally abusive (126). Internalized feelings of anger may also be experienced as self-criticism (127) and perpetuate depressive symptoms which may in turn increase risk for physical health problems particularly coronary heart disease (122). Capturing variation in sub-presentations of anger is therefore an important consideration in the development of perinatal mental health screening instruments for fathers.

## Conclusions

This study was the first to identify profiles of anger and depressive symptoms in men at the normative age for becoming a father. The five profiles that emerged were differentiated by symptom severity and by feelings of wanting to express anger physically. In a sub-sample of men with infants up to 18 months postpartum, compared to fathers in the Reference class, those with elevated symptoms reported lower social support and poorer co-parenting and those with the most severe symptoms reported problems bonding with their infant children. The results contribute to the current debate on the role of anger in male depressive symptomatology and are relevant to public health decision-making about paternal screening for postpartum mental health risk and for health care practitioner understanding of possible clinical presentations of men's depression. Most importantly, our results indicate a co-existing interpersonal risk of depression and anger that may be relevant to family safety, partner mental health and child developmental outcomes.

## Data Availability Statement

The raw data supporting the conclusions of this article will be made available by the authors, without undue reservation.

## Ethics Statement

The studies involving human participants were reviewed and approved by Deakin University, Faculty of Health, Human Ethics Advisory Group. The patients/participants provided their written informed consent to participate in this study.

## Author Contributions

JM, TH, LD, HS, CO, RF, TK, and JW: conceptualization and study design. CG, JM, GY, LG, LF, LD, and TH: specific study data experts. CG, GY, LG, and TH: statistical analyses. JM, LF, TH, and LD: literature review. JM, TH, CG, CO, GY, and LG: interpretation of results. JM and TH: drafting original introduction and conclusion. LG, CG, LF, TH, GY, and JM: drafting of original methods. LG, TH, GY, LF, and JM: drafting of original results. JM, CG, LF, TH, LG, GY, LD, HS, RF, TK, JW, JM, and CO: critical revision of manuscript and final acceptance of manuscript. All authors contributed to the article and approved the submitted version.

## Conflict of Interest

The authors declare that the research was conducted in the absence of any commercial or financial relationships that could be construed as a potential conflict of interest.
